# Histone Deacetylase Inhibitor Trichostatin A Ameliorated Endotoxin-Induced Neuroinflammation and Cognitive Dysfunction

**DOI:** 10.1155/2015/163140

**Published:** 2015-07-27

**Authors:** Chung-Hsi Hsing, Shih-Kai Hung, Yeong-Chang Chen, Tsui-Shan Wei, Ding-Ping Sun, Jhi-Joung Wang, Ching-Hua Yeh

**Affiliations:** ^1^Department of Medical Research, Chi Mei Medical Center, Tainan 710, Taiwan; ^2^Department of Anesthesiology, Chi Mei Medical Center, Tainan 710, Taiwan; ^3^Department of Anesthesiology, Taipei Medical University, Taipei 110, Taiwan; ^4^Department of Radiation Oncology, Buddhist Dalin Tzu Chi General Hospital, Chiayi 622, Taiwan; ^5^School of Medicine, Tzu Chi University, Hualien 970, Taiwan; ^6^Department of Surgery, Chi Mei Medical Center, Tainan 710, Taiwan; ^7^Department of Medicinal Botanicals and Health Applications, Da-Yeh University, Changhua 515, Taiwan

## Abstract

Excessive production of cytokines by microglia may cause cognitive dysfunction and long-lasting behavioral changes. Activating the peripheral innate immune system stimulates cytokine secretion in the central nervous system, which modulates cognitive function. Histone deacetylases (HDACs) modulate cytokine synthesis and release. Trichostatin A (TSA), an HDAC inhibitor, is documented to be anti-inflammatory and neuroprotective. We investigated whether TSA reduces lipopolysaccharide- (LPS-) induced neuroinflammation and cognitive dysfunction. ICR mice were first intraperitoneally (i.p.) injected with vehicle or TSA (0.3 mg/kg). One hour later, they were injected (i.p.) with saline or *Escherichia coli* LPS (1 mg/kg). We analyzed the food and water intake, body weight loss, and sucrose preference of the injected mice and then determined the microglia activation and inflammatory cytokine expression in the brains of LPS-treated mice and LPS-treated BV-2 microglial cells. In the TSA-pretreated mice, microglial activation was lower, anhedonia did not occur, and LPS-induced cognitive dysfunction (anorexia, weight loss, and social withdrawal) was attenuated. Moreover, mRNA expression of HDAC2, HDAC5, indoleamine 2,3-dioxygenase (IDO), TNF-*α*, MCP-1, and IL-1*β* in the brain of LPS-challenged mice and in the LPS-treated BV-2 microglial cells was lower. TSA diminished LPS-induced inflammatory responses in the mouse brain and modulated the cytokine-associated changes in cognitive function, which might be specifically related to reducing HDAC2 and HDAC5 expression.

## 1. Introduction

The loss of intellectual functions such as thinking, remembering, learning, and the activities of daily living is called* cognitive dysfunction*. The forms of cognitive dysfunction are seen in illnesses such as Alzheimer's and infectious diseases. In cognitive disorders, neurodegeneration and neuroinflammation have been implicated [1–3]. Neuroinflammation has been associated with altered neural circuitry after trauma and in neurodegenerative diseases, which suggests that the central nervous system (CNS) is involved in changing cognitive function by directly and indirectly affecting neurons [4, 5]. Communication between the immune system and the CNS is necessary and is a response to immune-induced immunological, physiological, and behavioral changes [6]. Proinflammatory cytokines and endotoxins induce a sickness behavior syndrome [7–9]. Proinflammatory cytokines such as tumor necrosis factor- (TNF-) *α*, interleukin- (IL-) 1*β*, and monocyte chemotactic protein- (MCP-) 1 in the brain are partially responsible for the behavioral symptoms of, for example, anorexia, social withdrawal, and anhedonia [10–13]. Activation of the peripheral innate immune system stimulates cytokine secretion in the CNS. These cytokines modulate the behavioral symptoms of sickness. The overexpression of inflammatory cytokines in the brain is associated with cognitive dysfunction, sickness behavior, and depression [14–16].

Microglia are one of two major innate types of immune cell in the CNS and mediate its immune responses, in particular to lipopolysaccharide (LPS). Activated microglia produce antigens, inflammatory cytokines, and phagocytosis. Limiting microglial activity is considered beneficial for neuroinflammatory changes [17–19].

Changes in gene expression in the brain are important in normal aging and in neurodegenerative disease-induced cognitive deficits. The posttranslational modification of various histones is involved in regulating and silencing gene expression. Histone acetylation is controlled by histone acetyl transferases (HATs), general transcription factors and histone deacetylases (HDACs), and suppressed gene transcription [20, 21]. HDACs and HDAC inhibitors modulate cytokine synthesis and release [22–24]. There are four classes of human HDACs [24]. Classes I and II are involved in brain disease [20]. HDAC inhibitors are both anticancer and anti-inflammatory agents. Several HDAC inhibitors have been reported as neuroprotective in rat models [25, 26]. Nonselective HDAC inhibitors such as trichostatin A (TSA) inhibit all Class I and Class II HDACs and induce histone acetylation. TSA regulates the cytokine expression in the LPS-mediated inflammatory response [22, 24]. Although TSA is anti-inflammatory [27], its role in LPS-induced neuroinflammation and cognitive dysfunction is unclear.

We investigated the effects of TSA in LPS-induced neuroinflammation and cognitive dysfunction and, in particular, whether TSA reduces that inflammation and dysfunction.

## 2. Materials and Methods

### 2.1. Animals

CD-1 (ICR) BR strain adult and juvenile mice (male, 8 weeks old, and 30–35 g) were purchased from BioLasco (Charles River Laboratories). All mice were housed individually in polypropylene cages and maintained in a temperature-controlled room (22 ± 2°C) on a 12 h light/dark cycle with* ad libitum* access to rodent chow and water except during the behavior-observation tests. At the end of each study, the mice were examined postmortem for signs of diseases such as splenomegaly or tumors. Data from mice determined to be unhealthy were excluded from analysis. All procedures were in accordance with the Taiwan National Institute of Health Guidelines for the Care and Use of Laboratory Animals and the Chi Mei Foundation Medical Center Animal Use Policy. Chi Mei Medical Center approved the animal care protocol for the experiments in this study.

### 2.2. Cell Culture

BV-2 microglial cell lines were cultured in RPMI 1640 supplemented with 10% fetal bovine serum (FBS) and antibiotics. The cells were maintained at 37°C in a humidified atmosphere and 5% CO_2_, and the growth medium was refreshed every two days until confluence. Cultures were washed twice and supplemented with medium containing experimental conditions.

### 2.3. Behavior Tests

Locomotor activity and social exploratory behavior were measured as previously described [10]. In brief, the mice were handled for 2 min each day for 5 days before experimentation to adapt them to routine handling. Tests were done during the dark phase (between 0800 and 1700) of the photoperiod under infrared lighting to aid video recording.

### 2.4. Locomotor Activity

The mice were maintained in their home cage, and locomotor activity was video-recorded during 3 min tests. On the video recordings, cages were divided into six identical rectangles and a trained observer who was blinded to experimental treatments determined the incidence of line crossing within 3 min.

### 2.5. Sucrose Preference

This test was done as previously described [9]. To assess sucrose preference, the mice were provided with two solutions, water or freshly prepared 2% sucrose, in 50 mL conical tubes with stoppers fitted with ball-type sipper tubes. Before the test, all the mice were adjusted to the two-bottle choice test to reduce their reactions to novelty and to ensure stability of the sucrose consumption baseline. To avoid any place preference, the relative location (left or right) of the sucrose bottle was changed whenever fluid intake was measured. Fluid consumption was measured by weighing bottles before and after each test session. The habituation period lasted until a stable sucrose intake level was reached. All the mice drank both the water and the 2% sucrose solution but preferred drinking the sucrose over the water (data not shown). On the day of the sucrose preference test, mice were deprived of fluid and food for 2 h before the test [11]. At the start of the dark phase of the photoperiod (0800), drinking water and the 2% sucrose solution were placed in the home cage for 9 h, 15 h, and 24 h, respectively. At the end of each testing period, the fluid content of the conical tubes was measured and sucrose preference was determined using the equation: sucrose intake/total fluid intake (water + sucrose intake) × 100% [8].

### 2.6. RNA Extraction and Reverse Transcriptase-Polymerase Chain Reaction (RT-PCR)

Total RNA from cultured BV-2 cells and from the cortex and hippocampus of the mice brains was extracted using Trizol reagent (Invitrogen, Carlsbad, CA, USA) as previously described [12]. Total mRNA (2 *μ*g) was synthesized to cDNA using reverse transcriptase kits (Clontech, BD Biosciences, Palo Alto, CA, USA) according to the manufacturer's protocol. Briefly, RNA samples were heated with RNase-free H_2_O and random primers at 70°C for 3 min and immediately cooled on ice. A mixture containing deoxyribonucleoside triphosphate (dNTP), 5× first-strand buffer, dithiothreitol (DTT), and MMLV (Moloney Murine Leukemia Virus) reverse transcriptase was subsequently gently mixed and then incubated at 42°C for 60 min. The reaction was then terminated by heating the mixture to 70°C for 15 min. RT-PCR was done on a thermal cycler (Applied Biosystems, Foster City, CA, USA) using 2x Taq DNA Polymerase Master Mix (Bioman Scientific Co., Jhonghe City, New Taipei City, Taiwan). The sequences of primers used in this experiment were as follows:

 mouse HDAC2:
   forward: 5′-GCG TAC AGT CAA GGA GGC GGC-3′;
   reverse: 5′-CCC CAG CAA CTG AAC CAC CCG-3′;
 mouse HDAC5:
   forward: 5′-CCT CAG CCT GGC CAC TGT GC-3′;
   reverse: 5′-TGT CCA CCC CAA TGC CCC CA-3′;
 mouse TNF-*α*:
   forward: 5′-GAG TGA CAA GCC TGT AGC CCA-3′;
   reverse: 5′-CCC TTC TCC AGC TGG AAG A-3′;
 mouse IL-1*β*:
   forward: 5′-GTG GCT GTG GAG AAG CTG TGG C-3′;
   reverse: 5′-TGG GTC CGA CAG CAC GAG GC-3′;
 mouse MCP-1:
   forward: 5′-AGG TCC CTG TCA TGC TTC TG-3′;
   reverse: 5′-GCT GCT GGT GAT CCT CTT GT-3′;
 mouse indoleamine 2,3-dioxygenase (IDO):
   forward: 5′-GAA GGA TCC TTG AAG ACC AC-3′;
   reverse: 5′-GAA GCT GCG ATT TCC ACC AA-3′;
 mouse *β*-actin transcript (internal control):
   forward: 5′-GGG AAT GGG TCA GAA GGA CT-3′;
   reverse: 5′-TTT GAT GTC ACG CAC GAT TT-3′.

*β*-actin, the housekeeping gene, was used to normalize all test genes. The data were analyzed using ImageJ software (version 1.41o) (National Institutes of Health, Bethesda, MD) (http://rsbweb.nih.gov/ij/), and results are expressed as fold differences.

### 2.7. Immunohistochemical Staining

Twenty-four hours after LPS administration, the mice were briefly anesthetized with isoflurane and then killed using cervical dislocation. The cortex and hippocampus of each mouse brain were fixed with 3.7% formaldehyde. All specimens were embedded in paraffin and sliced into 4 *μ*m thick sections. Sections were deparaffinized and then rehydrated, antigens were retrieved, and endogenous peroxidase activity was quenched using 3% hydrogen peroxide in PBS. After the sections had been blocked with an IHC blocking reagent (Background Sniper; Biocare Medical, Concord, CA, USA) for 1 h, they were incubated with anti-Iba1 (1 : 800 dilution) (Biocare Medical), anti-HDAC2 (1 : 200 dilution) or anti-HDAC5 (1 : 200 dilution) (Abcam, Cambridge, MA) rabbit anti-mouse antibodies in blocking reagent at 4°C overnight. Slides were then washed in PBS, incubated with species-specific biotinylated secondary antibody (1 : 200) for 30 min, washed with PBS again, amplified consecutively with Avidin horseradish peroxidase (HRP) (Vector Laboratories, Burlingame, CA, USA), and visualized by incubating them with 3,3′-diaminobenzidine tetrahydrochloride (Sigma-Aldrich, St. Louis, MO, USA). All slides were counterstained with hematoxylin (Mayer's; Thermo Shandon, Pittsburgh, PA, USA), dehydrated, and mounted. For negative Controls, the procedure omitted the primary antibody.

### 2.8. Enzyme-Linked Immunosorbent Assays (ELISAs)

Cell culture supernatants were collected and the levels of TNF-*α*, IL-1*β*, and MCP-1 (R&D Systems, Minneapolis, MN, USA) were measured using ELISA kits according to the manufacturer's instructions. All samples were run in triplicate. After the reaction, plates were washed and 100 *μ*L of* o*-phenylenediamine substrate (Sigma-Aldrich) was added to each well. Plates were incubated for 30 min at room temperature, after which 50 *μ*L of 4 N sulfuric acid was added to each well. The plates were read at 490 nm on a microplate reader (Spectra MAX 340PC), and the data were analyzed.

### 2.9. Western Blotting

Harvested cells were lysed with a buffer containing 1% Triton X-100, 50 mM of Tris (pH 7.5), 10 mM of ethylenediamine tetraacetic acid (EDTA), 0.02% of sodium azide, and a protease inhibitor cocktail (Roche Boehringer Mannheim Diagnostics, Mannheim, Germany). After one freeze-thaw cycle, cell lysates were centrifuged at 13,000 rpm for 20 min at 4°C. The lysates were boiled in sample buffer for 5 min. Protein samples (30 *μ*g/lane) were loaded on SDS-PAGE (sodium dodecyl sulfate polyacrylamide gel electrophoresis) and electrotransferred to a polyvinylidene fluoride (PVDF) membrane (Millipore, Billerica, MA, USA). Nonspecific bindings were blocked by incubating the membrane with 5% skim milk in Tris-buffered saline (TBS) containing 0.1% Tween 20 (TBST) for 2 h. The membranes were then hybridized with primary antibodies: inducible Iba1 (1 : 1000) (Biocare Medical), acetyl histone H3 (Lys9) (1 : 500) (Millipore), and *β*-actin (1 : 20000) (Sigma-Aldrich) at 4°C overnight. The membranes were then washed with 0.1% TBST and incubated with a 1 : 5000 dilution of species-specific HRP-conjugated secondary antibodies (Santa Cruz Biotechnology, Santa Cruz, CA, USA) at room temperature for 1 h. After they had been washed, the membranes were soaked in electrochemiluminescence (ECL) solution (PerkinElmer Life Sciences, Boston, MA, USA) for 1 min and then exposed to X-ray film (BioMax; Eastman Kodak, Rochester, NY, USA). The relative signal intensity was also quantified using ImageJ 1.41o.

### 2.10. Experimental Protocols

For* in vitro* studies, TSA (Cayman Chemical, Ann Arbor, MI, USA) and* E. coli* LPS (serotype O55:B5; Sigma-Aldrich) were prepared in isotonic PBS. BV-2 cells were washed and replenished with medium containing TSA (10 ng/mL). One hour later, LPS 50 ng/mL was added to the culture medium and incubated for 4 h. Total protein was collected from cell homogenates and determined using a kit (DC Protein Assay; Bio-Rad Laboratories, Hercules, CA, USA). Total RNA was isolated using Trizol reagent and the HDAC2, HDAC5, TNF-*α*, IL-1*β*, MCP-1, and IDO were assayed using RT-PCR.

For* in vivo* experiments, TSA and LPS were dissolved in pyrogen-free isotonic, sterile saline. In the first experiment, adult male ICR mice were injected (i.p.) with vehicle or TSA (0.3 mg/kg/body wt). One hour later, they were injected (i.p.) with saline or LPS (1 mg/kg) and asphyxiated 24 h later (*n* = 6 in each group). The dose of LPS was chosen because it caused a proinflammatory cytokine response in the brain and mild temporary sickness behavior [28]. Their brains were removed and dissected. The cortex was stored at −80°C and the hippocampus was fixed with 3.7% formaldehyde. Total RNA was isolated from these regions using Trizol. HDAC2, HDAC5, TNF-*α*, IL-1*β*, MCP-1, and IDO were assayed using RT-PCR.

In the second experiment, adult male ICR mice were treated with saline or TSA and then LPS, as described above (*n* = 6 in each group). Their motivation to engage in social behavior was determined immediately before the injection of saline or LPS, and again 2, 4, 8, and 24 h later. Body weight, food, and water intake were measured at each time point over the 24 h period. Anhedonia was evaluated using sucrose preference 26–47 h after the saline or LPS injection.

### 2.11. Statistical Analysis

Data were analyzed using SAS/STAT (SAS Institute, Cary, NC, USA) Generalized Linear Model (GENMOD) procedures. Data were subjected to Kruskal–Wallis ANOVA (analysis of variance) to determine significant main effects and interactions between main factors. When appropriate, a post hoc Tukey test was used to determine whether treatment means were significantly different from one another (*P* < 0.05). All data are means ± SEM (standard error of the mean).

## 3. Results

### 3.1. Effects of TSA on LPS-Treated Mice with Cognitive Dysfunction

**Figure 1:**
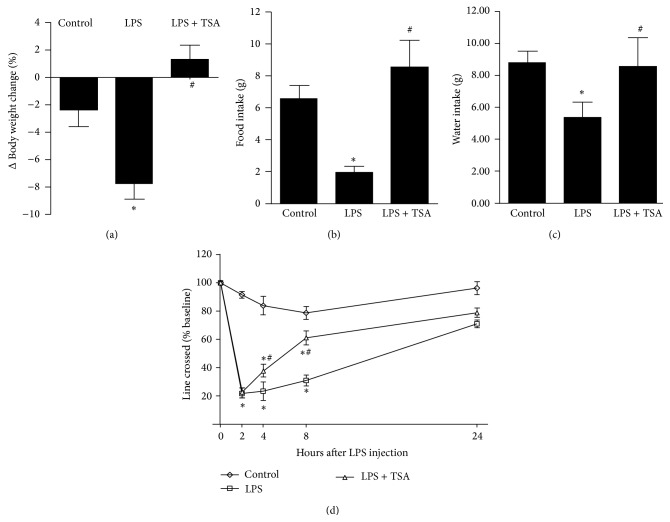
TSA facilitated the recovery from LPS-induced anorexia and increased line crossing (locomotor activity). Mice were pretreated with saline (Controls) or TSA (0.3 mg/kg) for 1 h and then intraperitoneally challenged with saline or* E. coli* LPS (1 mg/kg). (a) Body weight, (b) food intake, and (c) water intake were measured before an LPS injection and then again 24 h later. (d) Line crossing was measured before the LPS injection and then again 2, 4, 8, and 24 h later. Bars and graphs present means ± SEM (*n* = 6). ^∗^ *P* < 0.05 compared with the Control group; ^#^ *P* < 0.05 compared with the LPS-only group.

LPS-only-treated mice (LPS) lost significantly (*P* < 0.05) more body weight than did the saline-treated Controls and LPS + TSA-treated mice gained weight (Figure 1(a)). Food intake was significantly lower in the LPS group than in the Control group but significantly higher in the LPS + TSA group than in the LPS group (Figure 1(b)). Water intake was significantly lower in the LPS group than in the Control group but significantly higher in the LPS + TSA group than in the LPS group (Figure 1(c)). Eight hours after the mice in each group had been injected with the group treatment (saline [Control], LPS-only, or LPS + TSA) the number of line crossings in 3 min by each mouse was counted and compared with the number at baseline: there was a 20% decrease in saline-pretreated Controls, a 40% decrease in TSA-pretreated mice (*P* < 0.05), and an 80% decrease in LPS-only-treated mice (*P* < 0.05) (Figure 1(d)).

**Figure 2:**
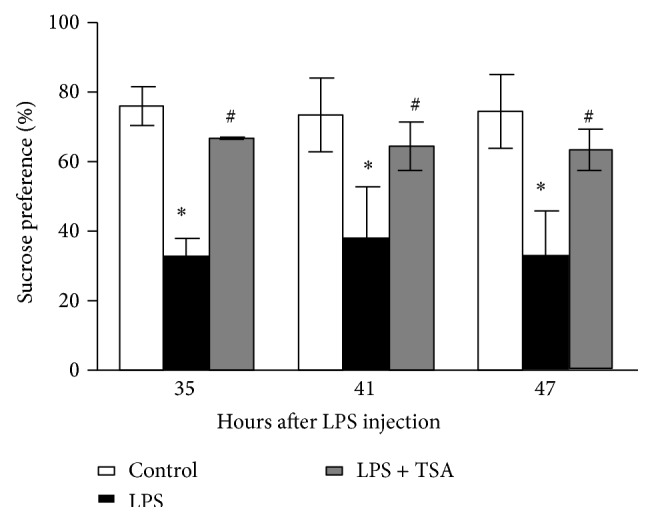
TSA inhibited LPS-induced anhedonia. Mice were pretreated with saline (Controls) or TSA (0.3 mg/kg) for 1 h and then intraperitoneally challenged with saline or* E. coli* LPS (1 mg/kg). The sucrose preference was determined 35 to 47 h after LPS injection. Bars represent the mean ± SEM (*n* = 6). ^∗^ *P* < 0.05 compared with the Control group; ^#^ *P* < 0.05 compared with the LPS-only group.

The effect of TSA on LPS-induced anhedonia was determined using a sucrose preference test. Between 35 and 47 h after the LPS challenge, sucrose preference was significantly lower in the LPS-only-treated group (Figure 2).

### 3.2. Neuroinflammation Was Significantly Lower in LPS + TSA-Treated Mice Than in LPS-Only-Treated Mice

**Figure 3:**
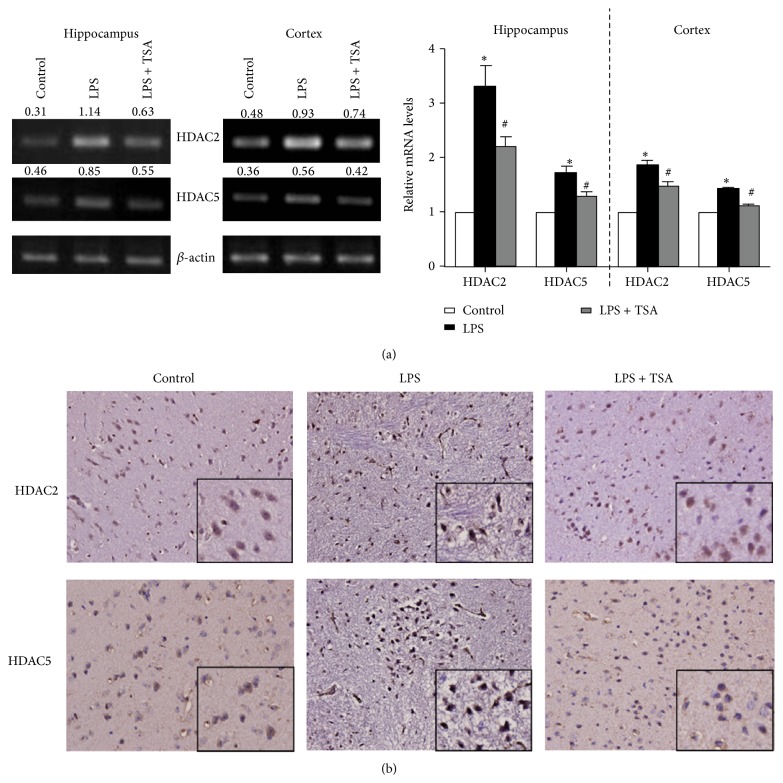
TSA attenuated LPS-induced HDAC2 and HDAC5 expression in the hippocampus and cortex of mice. Mice were pretreated with saline (Controls) or TSA (0.3 mg/kg) for 1 h and then intraperitoneally challenged with saline or* E. coli* LPS (1 mg/kg) (*n* = 6 in each group). (a) HDAC2 and HDAC5 mRNA were detected using RT-PCR. The right panel shows the quantification of mRNA expression from six independent experiments. ^∗^ *P* < 0.05 compared with the Control group; ^#^ *P* < 0.05 compared with the LPS-only group. (b) Immunohistochemical staining showed that HDAC2 and HDAC5 expression were increased in the cortex of mice after being LPS challenged, which were reduced in LPS + TSA mice.

**Figure 4:**
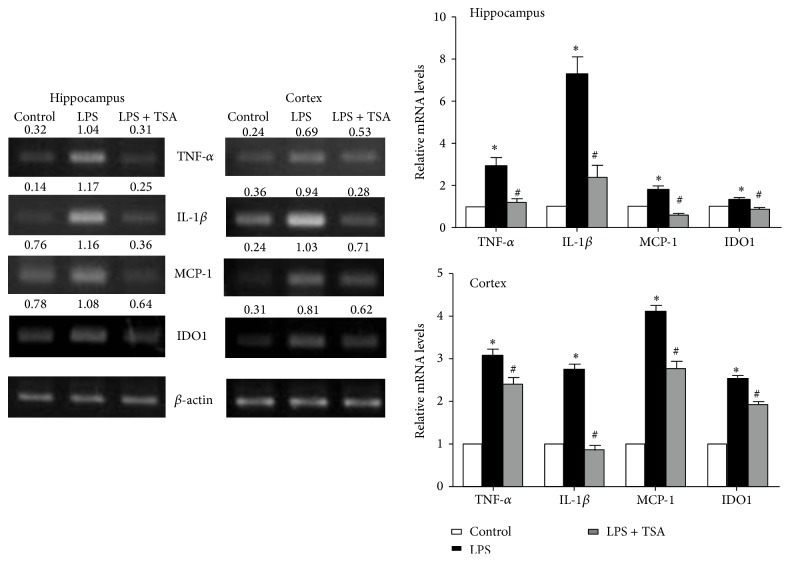
TSA attenuated LPS-induced cytokine mRNA expression in the hippocampus and cortex of mice. Mice were pretreated with saline (Controls) or TSA (0.3 mg/kg) for 1 h and then intraperitoneally challenged with saline or* E. coli* LPS (1 mg/kg) (*n* = 6 in each group). After 24 h they had been treated with LPS, TNF-*α*, IL-1*β*, MCP-1, and IDO mRNA expression in the hippocampus and cortex of mice determined using RT-PCR. The lower panel shows the quantification of mRNA expression from six independent experiments. ^∗^ *P* < 0.05 compared with the Control group; ^#^ *P* < 0.05 compared with the LPS-only group.

Mice pretreated with saline (Control) or TSA (0.3 mg/kg) were challenged with saline or LPS (1 mg/kg). Twenty-four hours later, the mice were killed and their cortex and hippocampus were collected. RT-PCR showed that their HDAC2 and HDAC5 mRNA expression levels were higher in the LPS-only-challenged mice. In the mice pretreated with TSA, HDAC2 and HDAC5 mRNA levels were significantly lower in both brain regions (Figure 3(a)). A histological examination showed that HDAC2 and HDAC5 levels were significantly higher in the cortex of LPS-only-challenged mice than in Control mice and in mice that had been pretreated with TSA (Figure 3(b)). HDAC inhibitors modulate cytokine synthesis and release [22–24]; we thus determined the effects of TSA on LPS-induced cytokines expression. After 24 h of LPS challenge, the expressions of TNF-*α*, IL-1*β*, MCP-1, and IDO mRNA in cortex and hippocampus were significantly higher than in Control mice. These increases were reduced in the mice pretreated with TSA (Figure 4).

### 3.3. TSA Inhibits LPS-Induced Microglia Activation

**Figure 5:**
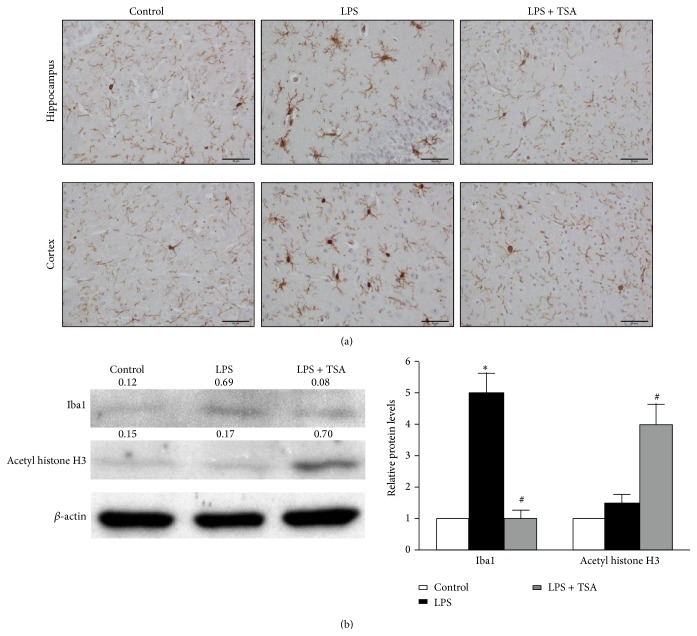
TSA reduced the LPS-induced activation of microglial cells in mice and BV-12 cells. (a) Mice were pretreated with saline (Controls) or TSA (0.3 mg/kg) for 1 h and then intraperitoneally challenged with saline or* E. coli* LPS (1 mg/kg). Immunohistochemistry staining shows that Iba1 expression in the hippocampus and the cortex of mice was higher after 24 h of LPS challenge than in Control and LPS + TSA mice. (b) One hour after BV-2 cells had been pretreated with TSA (10 ng/mL), they were treated with LPS. Four hours later, the protein was collected. Western blotting shows Iba1 and acetyl histone H3 expression in BV-2 cells. The right panel shows the quantification of protein expression from three independent experiments. ^∗^ *P* < 0.05 compared with the Control group; ^#^ *P* < 0.05 compared with the LPS-only group.

Microglia mediate cytokines expression in CNS and limiting microglial activity is considered beneficial for neuroinflammation [17–19]. We next determined the effects of TSA on LPS-induced microglia activation. A histological examination showed that Iba1 levels were significantly higher in the hippocampus and cortex of LPS-only-challenged mice, which were reduced in mice pretreated with TSA (Figure 5(a)). In the* in vitro* experiment, Iba1 protein expression was significantly higher in LPS-only-treated BV-2 microglial cells than in PBS Control and in LPS + TSA-treated cells (Figure 5(b)).

### 3.4. Cytokine Expression in LPS + TSA-Treated BV-2 Microglial Cells Was Significantly Lower Than in LPS-Only-Treated Cells

**Figure 6:**
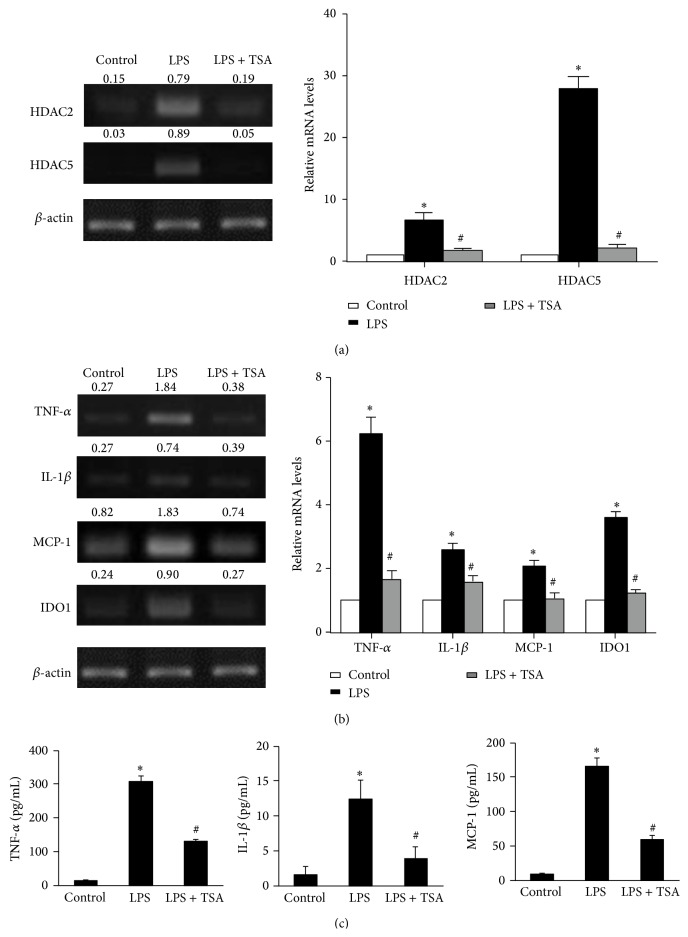
TSA reduced the LPS-induced mRNA expression levels of HDACs and cytokines in BV-2 cells. One hour after BV-2 cells had been pretreated with saline or TSA (10 ng/mL), they were treated with saline or LPS for 4 hours as indicated. (a) HDAC2 and HDAC5 and (b) TNF-*α*, IL-1*β*, MCP-1, and IDO mRNA expression were determined using RT-PCR. The right panel shows the quantification of mRNA expression from three independent experiments. (c) The protein levels of TNF-*α*, IL-1*β*, and MCP-1 in supernatants of the cultured BV-2 cells were determined using ELISA. Data are means ± SEM (*n* = 3 in each group). ^∗^ *P* < 0.05 compared with the Control group; ^#^ *P* < 0.05 compared with the LPS-only group.

In LPS-only-treated stimulated BV-2 cells, RT-PCR showed that HDAC2 and HDAC5 mRNA levels were significantly higher than in Control cells and in LPS + TSA-treated cells (Figure 6(a)). Moreover, TSA also induced acetyl histone H3 expression in LPS-treated BV-2 cells (Figure 5(b)). We also detected the expression levels of LPS-induced neuroinflammation mediators: TNF-*α*, IL-1*β*, MCP-1, and IDO. RT-PCR showed that TNF-*α*, IL-1*β*, MCP-1, and IDO mRNA were significantly higher in LPS-only-treated BV-2 cells than in Control cells and in LPS + TSA-treated cells (Figure 6(b)). We determined the protein levels of these cytokines' expression using ELISA. TNF-*α*, IL-1*β*, and MCP-1 concentrations in cultured supernatants of LPS-only-treated BV-2 cells were also significantly higher in LPS-only-treated BV-2 cells than in Control cells and in LPS + TSA-treated cells (Figure 6(c)).

## 4. Discussion

The present study showed that TSA effectively facilitated recovery from LPS-induced anorexia and inhibited anhedonia and increased the number of line crossings. Our* in vivo* and* in vitro* experiments showed that LPS increased HDAC2, HDAC5, TNF-*α*, IL-1*β*, MCP-1, and IDO expression and that TSA treatment decreased their expression. Previous studies [1–3, 10, 16] showed that neuroinflammation is associated with myriad complications: cognitive dysfunction, prolonged sickness behavior, and depressive-like behavior. After inducing anorexia and anhedonia via LPS administration, we used a locomotor activity and sucrose preference to assess behavior. Because of facility limit, we did not perform passive avoidance test (PAT) and Morris water maze (MWM) test to further evaluate mice behavior changes. Nevertheless, we determined the histology and gene expression changes which were related to neuroinflammation as well as cognitive dysfunction. All these changes were consistent in behavior experiments in this study.

Changes caused by histone acetylation are associated with cognitive function [29, 30]. HDAC inhibitors have been used to treat some brain disorders, for example, psychiatric diseases, cognitive impairment, and cognitive decline. HDAC inhibitors were neuroregenerative and neuroprotective in animal models of several neurological diseases [20]. Previous studies [30] showed that SAHA, an HDAC inhibitor, improved memory formation in a mouse model of neurodegeneration. The present study showed that TSA facilitated recovery from LPS-induced cognitive dysfunction.

HDAC Classes I and II are involved in brain disease [20]. HDAC1 has neuroprotective function but a previous study showed that it did not affect cognitive function in neuron-specific HDAC1 deletion mice [20, 30]. HDAC7 plays an important role in vascular endothelium but loss of HDAC7 does not affect brain disorders [20, 31]. Histone acetylation and memory formation ability were reduced in HDAC2-overexpressing mice (Class I group). Hippocampal overexpression of HDAC5 in mice (Class II group) induced depression-like behavior [30, 32]. HDAC inhibitors can facilitate learning and memory formation, which suggests that higher levels of histone acetylation are important for memory formation [33]. We found that TSA increased acetyl histone H3 expression in LPS-challenged microglial cells. HDAC2 and HDAC5 mRNA expression were also reduced by TSA in our* in vivo* and* in vitro* experiments.

In neurodegenerative or neuroinflammatory diseases, CNS has more activated microglia and higher levels of proinflammatory cytokines such as TNF-*α*, IL-1*β*, and IDO [13, 34]. In brain, the expression of IL-1*β* and other proinflammatory cytokines mRNAs are induced by systemically administering LPS, which affect cognitive dysfunction. Other studies [35, 36] have shown that IDO-mediated tryptophan metabolism is involved in neuroinflammatory disease. Inhibiting IDO activation prevents LPS-induced symptoms of depression, for example, anhedonia [16, 37]. We found that TSA reduced the levels of the proinflammatory cytokine TNF-*α*, IL-1*β*, and IDO expression in LPS-challenged mice brain and LPS-treated BV-2 cells.

HDAC activation modulates inflammation and immune cell activation [23]. LPS induces HDAC and proinflammatory cytokine expression in mouse microglia, and the cytokine expression is reduced by HDAC inhibitors [22]. The mechanism of HDAC and HDAC inhibitors on reducing proinflammatory cytokines in microglia is not fully understood. HDAC inhibitors are used for cancer therapy because they regulate gene transcription [38]. HDAC inhibitors induce or repress gene expression because acetylation markedly affects the function of nonhistone proteins, particularly reversibly acetylated transcription factors. The effects of HDAC inhibitors on inflammatory gene expression are based on the cell type and the stimulus [25, 27]. Previous studies report that TSA upregulates and downregulates different genes in the same cells. TSA increases LPS-stimulated IL-8 expression but inhibits IL-12 p40 expression in BEAS-2B cells [39]. HDAC inhibitors VPA and SAHA inhibit inflammatory cytokines but increase lymphocyte apoptosis and, dose-dependently, increase H3 acetylation [40]. We found that TSA reduced HDAC2 and HDAC5 mRNA expression and increased H3 acetylation in LPS-treated mice and cells. The TSA reduced LPS-induced proinflammatory cytokine expression. We also found that TSA treatment reduced the activation of microglia in LPS-challenged mice and BV-2 cells. Taking all our results together, TSA reduced HDAC and cytokine expression in LPS-treated mice and microglial cells. TSA facilitated recovery from LPS-induced cognitive dysfunction in mice. The effect of TSA on reducing LPS-induced neuroinflammation may be involved in the epigenetic regulation of cytokine expression. The regulatory mechanism that TSA uses to inhibit HDAC and cytokine expression requires additional investigation.

This study showed that TSA decreased LPS-induced sickness behavior symptoms (e.g., anorexia and anhedonia) and reduced microglia activation and neuroinflammatory cytokines production. These findings showed that TSA can be used to mitigate cytokine expression in the brain and beneficially affect mood, motivation, and behavior. We conclude that TSA can be a beneficial part of a pharmacological strategy to decrease infection-induced cognitive dysfunction and neuroinflammation.

## References

[1] Xu Y., Yan J., Zhou P. (2012). Neurotransmitter receptors and cognitive dysfunction in Alzheimer's disease and Parkinson's disease. *Progress in Neurobiology*.

[2] Wan Y., Xu J., Ma D., Zeng Y., Cibelli M., Maze M. (2007). Postoperative impairment of cognitive function in rats: a possible role for cytokine-mediated inflammation in the hippocampus. *Anesthesiology*.

[3] Terrando N., Monaco C., Ma D., Foxwell B. M. J., Feldmannc M., Maze M. (2010). Tumor necrosis factor-alpha triggers a cytokine cascade yielding postoperative cognitive decline. *Proceedings of the National Academy of Sciences of the United States of America*.

[4] Schlachetzki J. C. M., Hüll M. (2009). Microglial activation in Alzheimer's disease. *Current Alzheimer Research*.

[5] Morganti-Kossmann M. C., Satgunaseelan L., Bye N., Kossmann T. (2007). Modulation of immune response by head injury. *Injury*.

[6] Kelley K. W., Bluthé R. M., Dantzer R. (2003). Cytokine-induced sickness behavior. *Brain, Behavior, and Immunity*.

[7] Dantzer R., Kelley K. W. (2007). Twenty years of research on cytokine-induced sickness behavior. *Brain, Behavior, and Immunity*.

[8] Skalisz L. L., Beijamini V., Joca S. L., Vital M. A. B. F., Da Cunha C., Andreatini R. (2002). Evaluation of the face validity of reserpine administration as an animal model of depression-Parkinson's disease association. *Progress in Neuro-Psychopharmacology and Biological Psychiatry*.

[9] Frenois F., Moreau M., O'Connor J. (2007). Lipopolysaccharide induces delayed FosB/DeltaFosB immunostaining within the mouse extended amygdala, hippocampus and hypothalamus, that parallel the expression of depressive-like behavior. *Psychoneuroendocrinology*.

[10] Abraham J., Jang S., Godbout J. P. (2008). Aging sensitizes mice to behavioral deficits induced by central HIV-1 gp120. *Neurobiology of Aging*.

[11] Craft T. K. S., DeVries A. C. (2006). Role of IL-1 in poststroke depressive-like behavior in mice. *Biological Psychiatry*.

[12] Layé S., Parnet P., Goujon E., Dantzer R. (1994). Peripheral administration of lipopolysaccharide induces the expression of cytokine transcripts in the brain and pituitary of mice. *Molecular Brain Research*.

[13] Dantzer R., O'Connor J. C., Freund G. G., Johnson R. W., Kelley K. W. (2008). From inflammation to sickness and depression: when the immune system subjugates the brain. *Nature Reviews Neuroscience*.

[14] Chen J., Buchanan J. B., Sparkman N. L., Godbout J. P., Freund G. G., Johnson R. W. (2008). Neuroinflammation and disruption in working memory in aged mice after acute stimulation of the peripheral innate immune system. *Brain, Behavior, and Immunity*.

[15] Huang Y., Henry C. J., Dantzer R., Johnson R. W., Godbout J. P. (2008). Exaggerated sickness behavior and brain proinflammatory cytokine expression in aged mice in response to intracerebroventricular lipopolysaccharide. *Neurobiology of Aging*.

[16] Godbout J. P., Moreau M., Lestage J. (2008). Aging exacerbates depressive-like behavior in mice in response to activation of the peripheral innate immune system. *Neuropsychopharmacology*.

[17] Garden G. A., Möller T. (2006). Microglia biology in health and disease. *Journal of Neuroimmune Pharmacology*.

[18] Kraft A. D., Harry G. J. (2011). Features of microglia and neuroinflammation relevant to environmental exposure and neurotoxicity. *International Journal of Environmental Research and Public Health*.

[19] Saijo K., Glass C. K. (2011). Microglial cell origin and phenotypes in health and disease. *Nature Reviews Immunology*.

[20] Fischer A., Sananbenesi F., Mungenast A., Tsai L.-H. (2010). Targeting the correct HDAC(s) to treat cognitive disorders. *Trends in Pharmacological Sciences*.

[21] Peterson C. L., Laniel M.-A. (2004). Histones and histone modifications. *Current Biology*.

[22] Kannan V., Brouwer N., Hanisch U.-K., Regen T., Eggen B. J. L., Boddeke H. W. G. M. (2013). Histone deacetylase inhibitors suppress immune activation in primary mouse microglia. *Journal of Neuroscience Research*.

[23] Shuttleworth S. J., Bailey S. G., Townsend P. A. (2010). Histone Deacetylase inhibitors: new promise in the treatment of immune and inflammatory diseases. *Current Drug Targets*.

[24] Suh H.-S., Choi S., Khattar P., Choi N., Lee S. C. (2010). Histone deacetylase inhibitors suppress the expression of inflammatory and innate immune response genes in human microglia and astrocytes. *Journal of Neuroimmune Pharmacology*.

[25] Halili M. A., Andrews M. R., Labzin L. I. (2010). Differential effects of selective HDAC inhibitors on macrophage inflammatory responses to the Toll-like receptor 4 agonist LPS. *Journal of Leukocyte Biology*.

[26] Kim H. J., Rowe M., Ren M., Hong J. S., Chen P. S., Chuang D. M. (2007). Histone deacetylase inhibitors exhibit anti-inflammatory and neuroprotective effects in a rat permanent ischemic model of stroke: multiple mechanisms of action. *Journal of Pharmacology and Experimental Therapeutics*.

[27] Adcock I. M. (2007). HDAC inhibitors as anti-inflammatory agents. *British Journal of Pharmacology*.

[28] Bret-Dibat J.-L., Dantzer R. (2000). Cholecystokinin receptors do not mediate the suppression of food- motivated behavior by lipopolysaccharide and interleukin-1 beta in mice. *Physiology and Behavior*.

[29] Levenson J. M., O'Riordan K. J., Brown K. D., Trinh M. A., Molfese D. L., Sweatt J. D. (2004). Regulation of histone acetylation during memory formation in the hippocampus. *The Journal of Biological Chemistry*.

[30] Guan J.-S., Haggarty S. J., Giacometti E. (2009). HDAC2 negatively regulates memory formation and synaptic plasticity. *Nature*.

[31] Benn C. L., Butler R., Mariner L. (2009). Genetic knock-down of HDAC7 does not ameliorate disease pathogenesis in the R6/2 mouse model of Huntington's disease. *PLoS ONE*.

[32] Tsankova N. M., Berton O., Renthal W., Kumar A., Neve R. L., Nestler E. J. (2006). Sustained hippocampal chromatin regulation in a mouse model of depression and antidepressant action. *Nature Neuroscience*.

[33] Peleg S., Sananbenesi F., Zovoilis A. (2010). Altered histone acetylation is associated with age-dependent memory impairment in mice. *Science*.

[34] Rubio-Perez J. M., Morillas-Ruiz J. M. (2012). A review: inflammatory process in Alzheimer's disease, role of cytokines. *The Scientific World Journal*.

[35] van Dam A.-M., Brouns M., Louisse S., Berkenbosch F. (1992). Appearance of interleukin-1 in macrophages and in ramified microglia in the brain of endotoxin-treated rats: a pathway for the induction of non-specific symptoms of sickness?. *Brain Research*.

[36] Dantzer R. (2001). Cytokine-induced sickness behavior: where do we stand?. *Brain, Behavior, and Immunity*.

[37] O'Connor J. C., Lawson M. A., André C. (2009). Lipopolysaccharide-induced depressive-like behavior is mediated by indoleamine 2,3-dioxygenase activation in mice. *Molecular Psychiatry*.

[38] Walkinshaw D. R., Yang X.-J. (2008). Histone deacetylase inhibitors as novel anticancer therapeutics. *Current Oncology*.

[39] Iwata K., Tomita K., Sano H., Fujii Y., Yamasaki A., Shimizu E. (2002). Trichostatin A, a histone deacetylase inhibitor, down-regulates interleukin-12 transcription in SV-40-transformed lung epithelial cells. *Cellular Immunology*.

[40] Glauben R., Batra A., Fedke I. (2006). Histone hyperacetylation is associated with amelioration of experimental colitis in mice. *Journal of Immunology*.

